# Evaluation of left ventricular diastolic function by fractional area change using cine cardiovascular magnetic resonance: a feasibility study

**DOI:** 10.1186/1532-429X-15-87

**Published:** 2013-09-26

**Authors:** Satoshi Okayama, Tomoya Nakano, Shiro Uemura, Shinichi Fujimoto, Satoshi Somekawa, Makoto Watanabe, Tamio Nakajima, Yoshihiko Saito

**Affiliations:** 1 First Department of Internal Medicine, Nara Medical University, Nara, Japan; 2 Education Development Center, Nara Medical University, Nara, Japan; 3 Department of Cardiology, Hirai Hospital, Nara, Japan

**Keywords:** Cine, Diastolic function, Relaxation, Pseudonormal, Echocardiography, Color kinesis

## Abstract

**Background:**

Evaluation of left ventricular (LV) diastolic function is essential for the management of heart failure. We verified whether LV diastolic function could be evaluated by measuring the fractional area change (FAC) using cine cardiovascular magnetic resonance (CMR).

**Methods:**

We collected clinical data from 59 patients who underwent echocardiography and cine CMR. Normal, impaired relaxation, pseudonormal, and restrictive LV filling were observed in 15, 28, 11, and 5 patients, respectively. We calculated FAC during the first 30% of diastole (diastolic-index%) in the short-axis view, by tracing the contours on only three MR cine images.

**Results:**

The diastolic index was significantly lower (p < 0.0001) in patients with impaired relaxation (32.4 ± 7.5), pseudonormal filling (25.4 ± 5.6), and restrictive filling (9.5 ± 1.5) compared to those with normal diastolic function (67.7 ± 10.8), and the index decreased significantly with worsening of diastolic dysfunction. The diastolic index correlated positively with early diastolic mitral annular velocity measured by tissue Doppler imaging (r = 0.75, p < 0.0001), respectively.

**Conclusions:**

Measurement of FAC can be useful for the evaluation of LV diastolic function using cine CMR.

## Background

The accurate evaluation of left ventricular (LV) diastolic as well as systolic function is essential for the diagnosis and treatment of heart failure. Impaired LV diastolic function often precedes systolic dysfunction in heart failure and is observed at its earliest stage [1]. It is associated with increased end-diastolic pressure, dyspnoea, fatigue, and reduced exercise tolerance [2] and is closely associated with increased mortality [3]. Currently, echocardiography is the main noninvasive modality used for the evaluation of LV diastolic function [4].

On the other hand, cardiovascular magnetic resonance (CMR) has been widely used for the evaluation of left ventricular (LV) morphology and function due to its excellent image quality and lack of geometric assumptions. However, this modality is less popular for evaluating diastolic function despite the development of several relevant techniques, including the use of volumetric filling curves [5], phase-contrast imaging [6], tagging [7], and strain-encoded imaging [8]. This might be because these acquisition sequences or software for automatically measuring LV diastolic function are not yet applied generally [5,9].

Regional wall motion has also been evaluated using a color kinesis echocardiography system (Philips Medical Systems, Andover, MA, USA) [10]. This method can track and display endocardial motion by automatically tracing endocardial contours and evaluate LV diastolic function by measuring the degree of expansion during the first 30% of diastole in the short-axis view [11-15].

The purposes of this study were to apply the concepts of echocardiographic color kinesis (Echo-CK) to cine CMR and to determine whether measurement of LV fractional area change can enable easy evaluation of diastolic as well as systolic functions. Accordingly, cardiac function indices obtained from the measurement of LV fractional area change in cine CMR were compared to conventional echocardiographic measurements.

## Methods

### Subjects

We collected clinical data from consecutive patients who underwent cardiac MRI and transthoracic echocardiography for evaluation of LV diastolic function between January 1, 2010 and December 31, 2012 at Hirai Hospital. The exclusion criteria were as follows: (1) unfit for accurate evaluation by echocardiography, (2) arrhythmia, (3) difficulty in breath-holding, (4) pericardial disease, (5) moderate-to-severe aortic or mitral regurgitation assessed with color-flow Doppler echocardiography, and (6) mitral valve stenosis. Thus, we retrospectively evaluated 59 patients (41 men and 18 women, mean age 59.5 ± 18.9 years) with excellent-quality MR cine images. The present study conformed to the clinical research protocols approved by the institutional ethics committee of Hirai Hospital, in compliance with the 1975 Declaration of Helsinki.

### Echocardiography

LV systolic function was assessed by calculating ejection fraction (EF) with the Modified-Simpson method. LV and left atrial (LA) dimensions were measured by using M-mode echocardiography.

Next, diastolic function was assessed by transmitral and tissue Doppler imaging at the septal mitral annulus. Peak early (E) and late transmitral filling velocities (A), their ratio, the deceleration time (DCT) of peak E velocity, early diastolic mitral annular velocity (Ea), and E/Ea were measured. The subjects were classified into the following four groups according to the Recommendations for the Evaluation of Left Ventricular Diastolic Function by the American Society of Echocardiography [16]: (1) a normal diastolic function group with normal ejection fraction (≥60%) and increased Ea (≥8), which showed no echocardiographic evidence of cardiovascular diseases; (2) an impaired relaxation group with a reduced Ea (<8), a reduced E/A ratio (<0.8), and a long DCT (>200 ms); (3) a pseudonormal group with a reduced Ea (<8), an E/A ratio from 0.8 to 1.5, and no shortening of DCT (>160 ms); and (4) a restrictive group with a reduced Ea (<8), an increased E/A ratio (>2), and a shortened DCT (<160 ms).

### CMR Acquisition

Supine patients were examined at rest using a 1.5-T scanner (Avanto; Siemens, Erlangen, Germany) with an 8-element phased-array body coil. Cine images were acquired at a rate of 25 phases per cardiac cycle by using segmented ECG-triggered, steady-state, free-precession cine imaging with echo sharing (true fast imaging with steady-state, free precession [True FISP] sequence), from the same 8–10 contiguous short-axis planes with an 8-mm section thickness and an inter-slice gap of 2 mm, covering the entire LV from base to apex, as described previously [17].

### Evaluation of Cardiac Function by CMR

**Figure 1:**
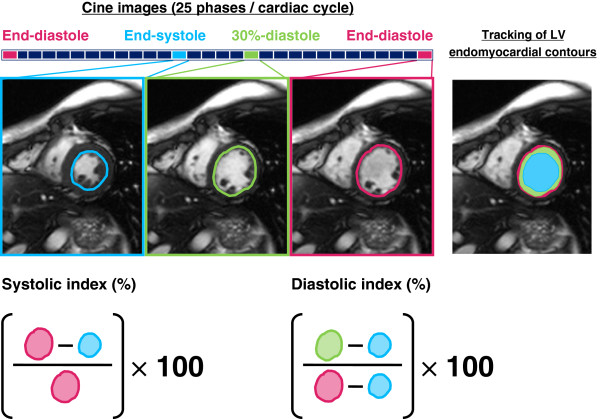
**Evaluation of left ventricular systolic and diastolic function by fractional area change in cine CMR.** End-diastolic and end-systolic phase are visually determined in datasets from short-axis cine images at mid papillary muscle, and phase number during 30% of diastole is calculated by multiplying total phase number during diastole by 0.3 and rounding up values. Left ventricular (LV) endocardial contours are then manually traced only on cine images of end-diastolic phase, end-systolic phase, and phase at 30% of diastole. The LV systolic fractional area change (systolic-index,%) and fractional area change during the first 30% of diastole (diastolic-index %) are calculated.

CMR data were analyzed by two experienced observers who were blinded to the clinical backgrounds of the participants, using integral software (Argus; Siemens). The concepts of Echo-CK were applied to short-axis cine image datasets at the level of the mid-papillary muscle. We visually determined the end-diastolic and end-systolic phases and calculated the phase number during 30% of diastole by multiplying the total phase number during diastole by 0.3 and rounding up the values. We manually traced LV endocardial contours on only cine images of the end-diastolic phase, end-systolic phase, and the phase at 30% of diastole and then calculated the systolic fractional area change (systolic-index, percent) and fractional area change during the first 30% of diastole (diastolic-index, percent) (Figure 1).

### Reproducibility of CMR evaluation of cardiac function by fractional area change

Intra-observer and inter-observer variability in the measurements of systolic and diastolic index was assessed in 20 randomly selected subjects. Two independent experienced observers who were blinded to each other’s findings calculated the systolic and diastolic indexes. One of the two observers reanalyzed the results after an interval of approximately one week.

### Statistical analysis

Data were statistically analyzed using GraphPad Prism (GraphPad software Inc., La Jolla, CA, USA) and are presented as means ± standard deviation. The differences between the groups were assessed using one-way analysis of variance followed by the post-hoc test. Systolic and diastolic indexes were compared to conventional echocardiographic cardiac function measurements by using linear regression analysis with Pearson’s correlation coefficient. Intra-observer and inter-observer variability in systolic and diastolic indexes were assessed by Bland–Altman analysis. A p value of <0.05 was considered statistically significant.

## Results

### Patient characteristics (Table 1)

**Table 1:** Clinical characteristics and echocardiographic variables in the four groups

	**Normal**	**Impaired relaxation**	**Pseudonormal**	**Restrictive**
Number	15	28	11	5
Age (years)	34.5 ± 15.3	71.1 ± 9.9	62.5 ± 10.5	63.0 ± 10.1
Male	10	21	6	4
Diagnosis		HHD 8	IHD 3	DHCM 1
		IHD 9	AS 1	DCM 4
		AS 1	HCM 3	
		HCM 3	DCM 3	
			Amyloidosis 1	
Echocardiographic variables				
LVEF (%)	65.9 ± 6.2	64.4 ± 6.5	56.9 ± 15.0	38.2 ± 10.0
LVDd (mm)	46.6 ± 4.4	46.8 ± 4.5	55.2 ± 7.5	66.4 ± 14.2
LAD (mm)	31.5 ± 3.7	38.1 ± 4.7	42.5 ± 5.5	47.2 ± 7.5
E/A	1.70 ± 0.77	0.69 ± 0.11	1.36 ± 0.22	2.24 ± 0.54
DCT (ms)	205.6 ± 34.9	234.8 ± 31.4	190.2 ± 23.5	143.2 ± 13.6
Ea (cm/s)	10.7 ± 1.9	6.3 ± 1.4	5.7 ± 1.2	4.9 ± 1.6
E/Ea	6.9 ± 1.0	9.5 ± 2.6	13.6 ± 3.3	20.4 ± 8.2

Values are mean ± standard deviation.

*HHD* hypertensive heart disease, *IHD* ischemic heart disease, *AS* aortic valve stenosis, *HCM* hypertrophic cardiomyopathy, *DCM* dilated cardiomyopathy, *DHCM* dilated-phase hypertrophic cardiomyopathy, *LVEF* left ventricular ejection fraction, *LVDd* left ventricular end-diastolic dimension, *LAD* left atrial dimension; E, peak early transmitral filling velocity; A, paek late transmitral filling velocity; DCT, deceleration time of peak E velocity; Ea, early diastolic mitral annular velocity.

The 59 subjects were divided into four groups on the basis of echocardiographic findings: (1) the normal diastolic function group, 15 patients; (2) the impaired relaxation group, 28 patients; (3) the pseudonormal group, 11 patients; and (4) the restrictive group, 5 patients. The age was significantly lower in the normal diastolic function group than in the other three groups (p < 0.0001). The LVEF was significantly lower in the restrictive group than in the other three groups (p < 0.01). The LVDd were similar between the normal diastolic function group and impaired relaxation group, and E/Ea increased significantly as diastolic dysfunction progressed from impaired relaxation to restrictive group (p < 0.05). The LAD was significantly smaller (p < 0.001) and the Ea significantly higher, in the normal diastolic function group compared to the other three groups (p < 0.001).

### LV systolic and diastolic index

**Figure 2:**
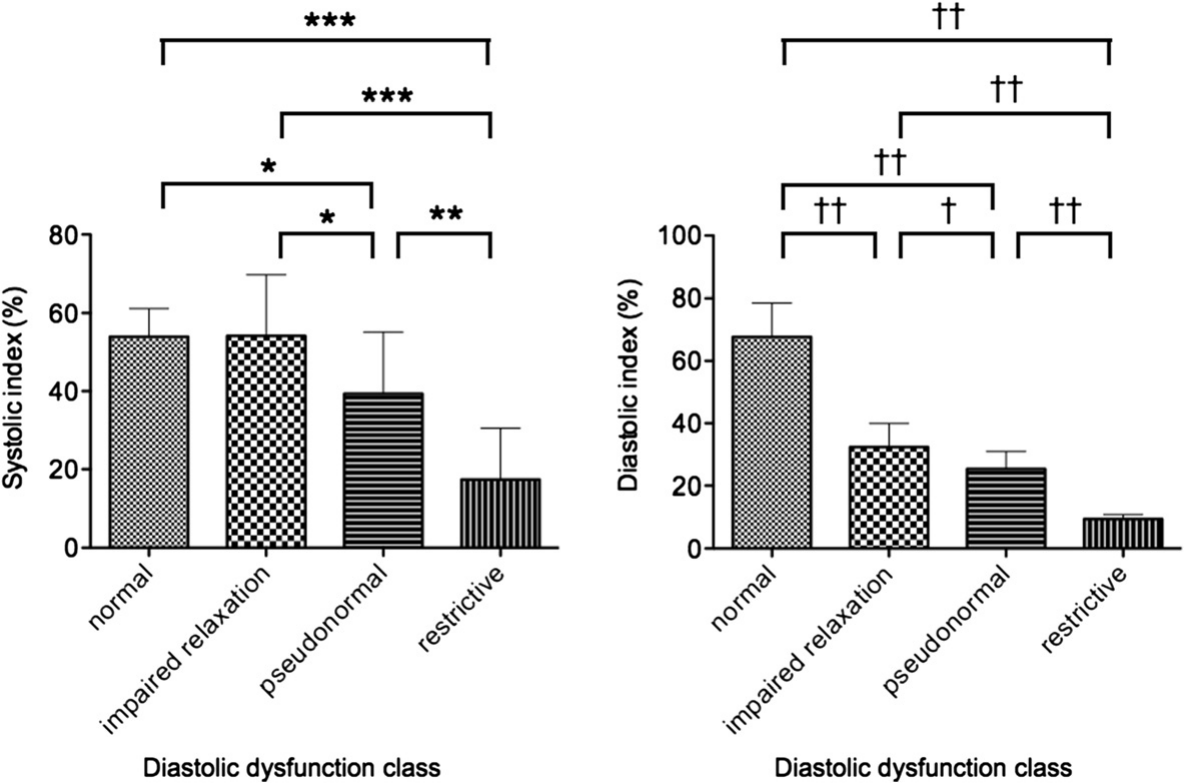
**Comparison of the left ventricular (LV) systolic and diastolic indexes among the three groups.** The systolic index was significantly lower in the restrictive group than in the other three groups, and significantly lower in the pseudonormal group than in the normal diastolic function group and the impaired relaxation group (*p < 0.05; **p < 0.01; ***p < 0.001). Whereas, the diastolic index was significantly higher in the normal diastolic function group compared to the three groups of diastolic dysfunction, and showed a significant reduction with the worsening of diastolic dysfunction (†p < 0.05; ††p < 0.001).

Figure 2 shows the comparison of systolic and diastolic indexes among the four groups. The LV systolic index was 54.0 ± 7.2 in the normal diastolic function group, 54.2 ± 15.6 in the impaired relaxation group, 39.4 ± 15.8 in the pseudonormal group, and 17.4 ± 13.1 in the restrictive group. The systolic index was significantly lower in the restrictive group than in the other three groups, and significantly lower in the pseudonormal group than in the normal diastolic function group and the impaired relaxation group. The LV diastolic index was 67.7 ± 10.8 in the normal diastolic function group, 32.4 ± 7.5 in the impaired relaxation group, 25.4 ± 5.6 in the pseudonormal group, and 9.5 ± 1.5 in the restrictive group. The diastolic index was significantly higher in the normal diastolic function group compared to the three groups of diastolic dysfunction and showed a significant reduction with the worsening of diastolic dysfunction.

**Figure 3:**
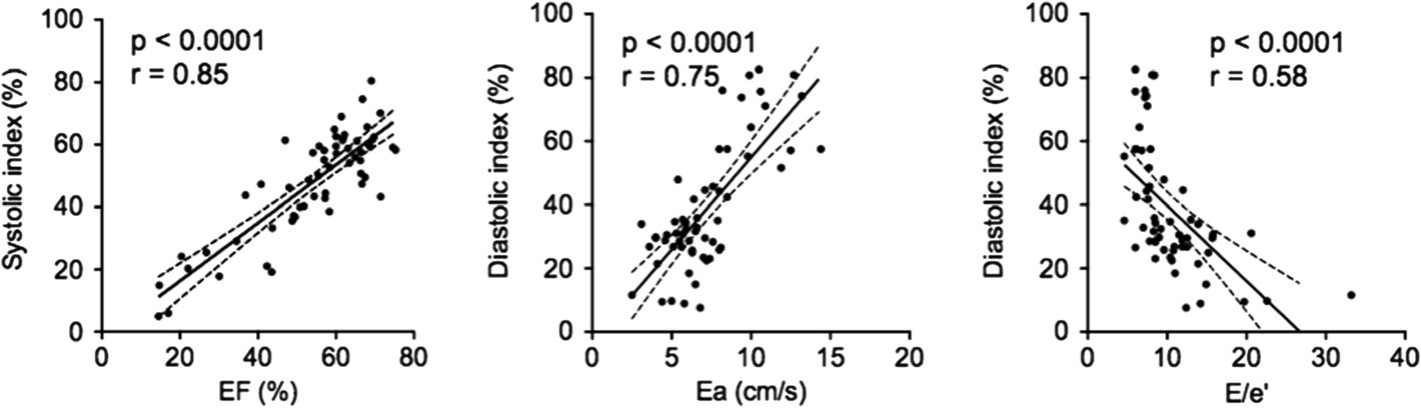
**The association between the left ventricular (LV) systolic and diastolic indexes and conventional echocardiography cardiac function measurements.** The LV systolic index well positively correlated with ejection fraction (EF), and the diastolic index moderately positively correlated with early diastolic mitral annular velocity (Ea) and negatively with the ratio of peak early filling velocity divided by Ea (E/Ea).

Figure 3 shows the association between the LV systolic and diastolic indexes and conventional echocardiographic cardiac function measurements. The systolic index showed good positive correlation with EF (r = 0.85, p < 0.0001), whereas the diastolic index showed moderate positive correlation with Ea (r = 0.75, p < 0.0001) and negative correlation with E/Ea (r = 0.58, p < 0.0001), but no correlation with E/A (p = 0.14) and DCT (p = 0.35). The reduction of diastolic index was closely associated with the progression of LV diastolic dysfunction.

The intra-observer reproducibility of the systolic and diastolic indexes was 0.88 ± 4.10% and −0.12 ± 5.62%, respectively, while the corresponding inter-observer reproducibility was 3.40 ± 5.90% and 3.03 ± 11.1%.

## Discussion

This is the first study to show that the concepts of Echo-CK could be applied to MR cine imaging, and that LV diastolic and systolic function could be evaluated by the measurement of fractional area change. The degree of LV systolic and diastolic dysfunction evaluated by fractional area change in cine CMR correlated well with that determined by echocardiography. Evaluation of cardiac function by fractional area change is not new; however, it is considered to be useful because of the availability of a few simple techniques for the evaluation of diastolic function using CMR.

The original Echo-CK automatically traces the endocardial contours based on acoustic quantitation and is thus, difficult to apply to low-quality images due to a poor echo-window. In contrast, the quality of cine CMR is always excellent in individuals without arrhythmia who have no difficulty in breath-holding, and thus, we could easily apply the concepts of Echo-CK to cine image datasets obtained for all our study patients.

We defined the diastolic index as fractional area change during the first 30% of the diastole for the following reasons, as explained by the echocardiographic studies [11-15]. LV diastolic function consists of relaxation and filling, and the diastolic phase consists of an isovolumic relaxation phase and a filling phase in which blood flows from the LA to the LV. The isovolumic relaxation phase is a highly energy-demanding process and can be delayed by a slight deficit of myocardial energy. The fractional area change during the first 30% of diastole, mainly representing early regional LV relaxation, which occurs during isovolumic relaxation and early diastolic filling, is thus considered to be a more sensitive indicator of diastolic dysfunction. In this study, the systolic and diastolic indexes derived by CMR were 54.0 ± 7.2 and 67.7 ± 10.8 in patients with normal diastolic function, respectively. The diastolic index could differentiate between the normal and pseudonormalized pattern of LV inflow and classify the severity of diastolic dysfunction. The diastolic index correlated well with Ea and E/Ea, but not with E/A and DCT on echocardiography. We consider that the diastolic index reflects more directly the early diastolic myocardial motion than the transmitral flow determined by multiple parameters, such as filling pressure, LV elastic recoil, myocardial relaxation, and atrial and chamber compliance. These results agree with those of the echocardiographic studies [11-15]. Harada et al. concluded that the original Echo-CK can quantitatively evaluate the LV diastolic function by enabling the detection of regional LV delayed relaxation.

Evaluation of LV diastolic function by fractional area change in cine CMR has two advantages. First, it does not entail the expense of development of an acquisition sequence or post-processing software, and LV diastolic function can be easily evaluated by the existing equipment. Second, LV diastolic function can be retrospectively evaluated if prior cine image datasets are stored. In contrast, this method has inherent disadvantages, and four disadvantages similar to the original Echo-CK [10,11]. First, manual tracing of the LV endocardial contours in cine CMR images may limit the variability in the spatial and temporal resolution, which influences reproducibility to some extent. Therefore, we believe that the automatic tracing software needs to be developed to resolve this non-negligible problem. Second, the method simply evaluates the motion of an endocardial contour in a slice and ignores the influence of cardiac translation and/or rotation. However, the influence is considered to be relatively small because this method evaluates endocardial motion rather than wall thickening. Third, the method assesses regional, not global cardiac function. Fourth, clear images with minimum noise are needed. Naturally, a more detailed evaluation of cardiac function requires not only the measurement of fractional area change but echocardiography or CMR studies such as volumetric filling curves, phase-contrast imaging, tagging, and strain-encoded imaging.

The present study has the following four limitations. First, we retrospectively evaluated the data from a relatively small sample cohort at a single center. Second, a hemodynamic study was not performed. Third, the original Echo-CK method was not employed. Fourth, LV fractional area change was not evaluated in vertical or horizontal long-axis views. The changes in the vertical or horizontal long-axis views might not be similar to those in the short-axis view because the heart has a complex three-dimensional structure consisting of myocardial fibers oriented in different directions [18]. Evaluation of LV function by fractional area change in cine CMR should be prospectively tested in many more patients with several cardiac diseases at several centers.

## Conclusions

The concepts of Echo-CK were applied to cine CMR, and the measurement of LV fractional area change on cine CMR can be employed as a screening tool to detect diastolic as well as systolic dysfunction.

## Consent

Written informed consent could not be obtained from the subjects for the publication of this report and any accompanying images because the study was retrospectively designed and performed. However, the identity of the subjects are protected in this report.

## Abbreviations

CMR: Cardiovascular magnetic resonance; DCT: Deceleration time; EF: Ejection fraction; FAC: Fractional area change; LA: Left atrium; LV: Left ventricular; MR: Magnetic resonance.

## Competing interests

The authors declare that they have no competing interests.

## Authors’ contributions

SO; Analysis, and drafting the manuscript or revising it. TN; Analysis and interpretation of data. SU; Helping to draft the manuscript. SF; Contributions to conception and design. SS; Statistics. MW; Acquisition of data. TN; Acquisition of data. YS; Contributions to conception and design. All authors read and approved the final manuscript.
